# Epigenetic Evaluation of the *TBX20* Gene and Environmental Risk Factors in Mexican Paediatric Patients with Congenital Septal Defects

**DOI:** 10.3390/cells12040586

**Published:** 2023-02-11

**Authors:** Esbeidy García-Flores, Juan Calderón-Colmenero, Verónica Marusa Borgonio-Cuadra, Juan Pablo Sandoval, José Antonio García-Montes, Benny Giovanni Cazarín-Santos, Antonio Miranda-Duarte, Armando Gamboa-Domínguez, José Manuel Rodríguez-Pérez, Nonanzit Pérez-Hernández

**Affiliations:** 1 Departamento de Biología Molecular, Instituto Nacional de Cardiología Ignacio Chávez, Ciudad de México 14080, Mexico; 2 Sección de Estudios de Posgrado e Investigación, Escuela Superior de Medicina, Instituto Politécnico Nacional, Ciudad de México 11340, Mexico; 3 Departamento de Cardiología Pediátrica, Instituto Nacional de Cardiología Ignacio Chávez, Ciudad de México 14080, Mexico; 4 Departamento de Medicina Genómica, Instituto Nacional de Rehabilitación Luis Guillermo Ibarra Ibarra, Ciudad de México 14389, Mexico; 5 Laboratorio de Hemodinámica e Intervención en Cardiopatías Congénitas, Instituto Nacional de Cardiología Ignacio Chávez, Ciudad de México 14080, Mexico; 6 Departamento de Patología, Instituto Nacional de Ciencias Médicas y de la Nutrición Salvador Zubirán, Ciudad de México 14080, Mexico

**Keywords:** *TBX20* gene, promotor region, epigenetics, DNA methylation, in silico analysis, congenital septal defects, environmental risk factors

## Abstract

The *TBX20* gene has a key role during cardiogenesis, and it has been related to epigenetic mechanisms in congenital heart disease (CHD). The purpose of this study was to assess the association between DNA methylation status and congenital septal defects. The DNA methylation of seven CpG sites in the *TBX20* gene promoter was analyzed through pyrosequencing as a quantitative method in 48 patients with congenital septal defects and 104 individuals with patent ductus arteriosus (PDA). The average methylation was higher in patients than in PDA (*p* < 0.001). High methylation levels were associated with a higher risk of congenital septal defects (OR = 4.59, 95% CI = 1.57–13.44, *p* = 0.005). The ROC curve analysis indicated that methylation of the *TBX20* gene could be considered a risk marker for congenital septal defects (AUC = 0.682; 95% CI = 0.58–0.77; *p* < 0.001). The analysis of environmental risk factors in patients with septal defects and PDA showed an association between the consumption of vitamins (OR = 0.10; 95% CI = 0.01–0.98; *p* = 0.048) and maternal infections (OR = 3.10; 95% CI = 1.26–7.60; *p* = 0.013). These results suggest that differences in DNA methylation of the *TBX20* gene can be associated with septal defects.

## 1. Introduction

During embryonic development, the heart is the first organ to be developed through cardiogenesis, which is a highly complex process that requires the participation of different genes and transcriptional pathways. When a dysregulation in the gene expression exists due to genetic mutations or epigenetic alterations, it could affect heart development and lead to congenital heart disease (CHD) [1,2]. CHDs are the most frequent birth defects worldwide, with a prevalence of 1% in the general population [3]. In Mexico, however, the prevalence varies from region to region, with reports from 2.1 to 12.3 per 1000 live births [4]. It is known that the etiology of CHDs is multifactorial, with genetic and environmental components [5,6]. Regarding the genetic component, several genes are associated with the pathogenesis of CHD, mainly the transcription factors involved with heart development, such as *NKX2.5*, *GATA4*, *TBX1*, *TBX5*, *TBX20*, *LINE1,* and *HAND1* [7,8]. The transcriptional regulation of these genes could be coordinated by epigenetic mechanisms [9,10].

In this regard, *TBX20* belongs to the *T-box* gene family. This family plays a very important role in cardiac development. *TBX20* is a member of the *Tbx1* subfamily that actively participates in the early cardiac progenitor, myocardium, endocardium, precursors of cardiac valves, and atrioventricular septum [11]. *TBX20* has a key role in the regulation and proliferation of cardiac cells, and it modulates the interaction of others transcription factors (*NKX2.5*, *GATA4*, *GATA5*, and *TBX5*) involved in the cardiogenesis process [12,13,14]. Various studies of mutations in this family have been associated with congenital cardiac malformations. In particular, mutations in the *TBX20* gene have been related to septation defects, valvulogenesis, and cardiomyopathy [15,16]. A complete lack of function of the *TBX20* gene results in abnormal regulation of cardiac gene expression that may disturb the formation of cardiac progenitor cells associated with the presence of CHDs [11,17]. Moreover, recent studies have demonstrated the association of epigenetic mechanisms, particularly the DNA methylation of the *TBX20* gene in some types of CHDs [18,19]. Regarding the environmental component, some studies have evaluated the effects of being exposed to environmental factors during pregnancy, how these factors modify fetal growth, and if they could be risk factors for CHDs [20,21]. These studies have reported an association between maternal infections, alcohol intake, cigarette smoking, medication consumption, and folic acid intake, among others, with CHDs [18,22,23,24].

Nonetheless, the epigenetic impact of the *TBX20* gene on the development of congenital septal defects has been scarcely studied. Therefore, our research study towards to evaluate the key role of DNA methylation as an epigenetic phenomenon on the *TBX20* gene, this is considering that it is a gene with a regulatory function for the opportune expression of other cardiac genes during the first weeks for proper heart formation. Therefore, we aimed to evaluate the association of DNA methylation levels of *TBX20* gene promoter and the exposure to environmental risk factors with the risk of developing CHD, specifically, septal defects.

We also performed bioinformatic analysis to identify potential binding sites for the transcription factor in the *TBX20* gene promoter; moreover, we analyzed the interaction between *TBX20* gene methylation with environmental factors using the multifactor dimensionality reduction (MDR) algorithm. Lastly, we performed a protein-protein interaction (PPI) network to determine the possible regulation of this gene in CHDs.

## 2. Materials and Methods

### 2.1. Study Population

An observational, cross-sectional, and comparative study was performed in which individuals with CHD who attended the Paediatric Cardiology Department at the National Institute of Cardiology Ignacio Chavez in Mexico City were recruited. The study was in compliance with the Helsinki Declaration and was approved by the Research and Ethics Committees of the National Institute of Cardiology Ignacio Chavez (Register No. 19-1138). Written informed consent was obtained from each participant and/or their parents.

The study included a total of 152 participants from different regions in Mexico. The study group was formed by 48 patients with septal defects, and the comparison group was formed by 104 patients with PDA. Two expert genetic specialists detailed a clinical and phenotypic examination of every participant with the purpose of discarding any syndromic associated with CHD.

The inclusion criteria for patients with septal defects were newborn to eighteen-year-old individuals; of both sexes, with a diagnosis of the ventricular septal defect (VSD) or atrial septal defect (ASD) assessed by echocardiography characteristics, chest X-rays, and clinical and physical examinations. The criteria for diagnosis of septal defects (VSD and ASD) were based on the International Paediatric and Congenital Cardiac Code (IPCCC) [25], which classifies VSD and ASD as congenital malformations of the septum. The presence of VSD was defined as a congenital heart malformation in which there is a pathway or hole between the ventricular chambers [26]. ASD was defined as a malformation in the atrial septum that allows direct communication between the atria [27].

The comparison group included individuals with patent ductus arteriosus (PDA), considering that ductus arteriosus is a normal and necessary structure in fetal circulation, and closure of the ductus arteriosus occurs functionally at birth. The ductus arteriosus is not properly a heart defect, provided its failure to close during the newborn period is primarily due to physiological events involving Prostaglandin E2 and oxygen pressure [28]. The inclusion criteria for PDA were newborn to eighteen-year-old individuals; of both sexes; with a diagnosis of PDA based on IPCCC criteria [25].

In addition, the inclusion criteria for the study groups are that they present an isolated diagnosis of any of the three clinical entities evaluated, and the exclusion criteria for the study groups were patients with another diagnosis of heart defects and the presence of infectious heart disease.

Additionally, we collected data regarding the presence of environmental risk factors during pregnancy in mothers of patients with CHD. The environmental factors evaluated were: exposure to pollutants, consumption of vitamins, diseases during pregnancy, maternal infections, maternal addictions, and medication consumption.

### 2.2. DNA Extraction and Bisulfite Treatment

First, DNA was isolated from 3–5 mL of peripheral blood samples employing the kit QIAamp^®^ DSP DNABlood Mini (QIAGEN, Hilden, Germany); the evaluation of purity and concentration of the isolated DNA was determined with a Nanodrop 2000 Spectrophotometer (Thermo Fisher Scientific, Waltham, MA, USA). Genomic DNA was stored at −20 °C. A total of 1000 ng of DNA was utilized in the bisulfite conversion treatment using the Epitect Bisulfite Conversion Kit (QIAGEN, Hilden, Germany) following the manufacturer’s instructions. In the final process, each sample of bisulfite-converted DNA was suspended in 60 µL of elution buffer for the pyrosequencing technique.

### 2.3. Quantitative DNA Methylation Analysis

The chr7:35252863-35256864 region corresponds to a CpG island of the *TBX20* gene promoter, and it was selected for the DNA methylation quantification. The region analyzed: CGCGTCGCGTGGTTACCGTAGAGCTCCGCGC included seven CpG sites localized from base −751 to −782 to the transcription start site (TSS). The PCR technique was performed using the PyroMark PCR Kit (QIAGEN, Hilden, Germany). It required a volume of 25 µL, the incorporation of 25 ng of bisulfite-converted DNA, 12.5 µL of PyroMark PCR master mix 2X, 0.2 µM forward and reverse primers, and 2.5 µL of Coral load concentrate 10X. The PCR mixture was pre-heated for 15 min at 95 °C, then incubated for 45 cycles at 95 °C for 30 s, 56 °C for 30 s, and 72 °C for 60 s, followed by 72 °C for 10 min. The PCR final product (amplicon length 227 bp) was observed using 1.5% agarose gels with ethidium bromide staining. For pyrosequencing, a pre-designed assay of *TBX20* gene: Hs_TBX20_02 PM PyroMark CpG was used (GeneGlobe ID: PM00030919), commercially available in the QIAGEN, website (https://www.qiagen.com/us). This experimental strategy was performed to quantify the methylation levels of the CpG site expressed in percentage through PyroMark Q24 equipment (QIAGEN, Hilden, Germany). The PCR products previously obtained were utilized to form single-stranded DNA templates incorporated with the pyrosequencing primer following the manufacturer’s protocol. To validate the quality control of the procedure, each DNA sample evaluated for pyrosequencing included a negative control, as well as EpiTect DNA unmethylated and methylated controls (QIAGEN Cat. Num. 59695). The PyroMark Q24 Advanced Software V.3.0.0 evaluated the appropriate bisulfite conversion process; this program also allowed us to visualize and analyze the methylation levels of each CpG site expressed as percentages through a pyrogram.

### 2.4. Evaluation of Interaction between TBX20 Gene Methylation with Environmental Factors Using Multifactor Dimensionality Reduction (MDR) Algorithm

To analyze the interaction between *TBX20* gene methylation with environmental factors, the multifactor dimensionality reduction (MDR) algorithm was developed. The MDR 3.0.2 Software is free and available at epistasis.org, and the full procedure can be reviewed elsewhere [29,30]. The algorithm generates the interaction models showing their training and testing accuracy (i.e., the proportion of subjects that are grouped correctly according to their disease status: septal defects or PDA) and the cross-validation consistency (CVC). The best model has the highest CVC and testing accuracy values since this model shows more consistent results and is the most likely to generalize to independent data. Afterward, interaction models showing the highest CVC and testing accuracy values were tested by 1000 folds permutation tests and Chi-squared test at an α significance levels <0.05.

### 2.5. In Silico Analysis for Prediction of Transcription Factor Bindings Sites

To identify potential binding sites for transcription factors in the sequence analyzed by pyrosequencing, an in silico analysis was performed with the online bioinformatic tools MatInspector, website (https://www.genomatix.de/online_help/help_matinspector/matinspector_help.html accessed on 18 May 2022), and PROMO version 3.0.2, website (http://alggen.lsi.upc.es/cgi-bin/promo_v3/promo/promoinit.cgi?dirDB=TF_8.3 accessed on 9 June 2022).

### 2.6. Screening of Cardiac Genes Associated with CHD by Protein-Protein Interaction (PPI) Network

To identify PPI networks of the TBX20 protein, a search was performed with the STRING online database Version 11.5, website (https://stringdb.org/cgi/input?sessionId=bQw4wgfdWNSS&input_page_show_search=on (accessed on 29 June 2022). This analysis included interactions with high confidence >0.700; thus, the interactions obtained were significant.

### 2.7. Statistical Analysis

Data normality tests were performed, data of quantitative variables with non-normal distribution were presented as medians and interquartile ranges, and for these comparisons, a Mann-Whitney U test was applied. For qualitative variables, the Chi-squared test was performed. To determine differences in methylation levels by sex between septal defect patients and the PDA group, a Mann-Whitney U test was applied. To determine the association of environmental factors with the presence of CHD, odds ratio values and its 95% confidence intervals were obtained by univariate logistic regression. Regarding the distribution of methylation levels between the study groups, the levels were categorized as quartiles, and the lowest quartile was used as the reference group to calculate the risk of developing septal defects. The Spearman correlation analysis was performed to evaluate the correlation between age and methylation levels of the *TBX20* gene promoter among the study groups. To investigate if the methylation levels of the *TBX20* gene promoter could be used as a predictor test/value for the presence of septal defects, a receiver operator characteristic (ROC) curve analysis was performed, and the area under the curve (AUC) with their confidence intervals was calculated. All statistical analyses were two-tailed, and a *p*-value < 0.05 was considered statistically significant. Data were analyzed using GraphPad Prism Software 6.01 (GraphPad Software, La Jolla, CA, USA) and the SPSS 22.0 Software (SPSS Inc., Chicago, IL, USA).

## 3. Results

The septal defects group included 48 non-syndromic paediatric patients (52.1% females, 47.9% males) with a median age of 7 years [IQR: 4–11.7]; the comparison group included 104 non-syndromic PDA paediatric individuals (69.2% females, 30.8% males) with a median age of 3 years [IQR: 2–6]. These general characteristics of the study population are presented in Table 1.

### 3.1. Frequency of Environmental Risk Factors in the Study Population

The presence of environmental risk factors during pregnancy in mothers of individuals with CHD showed significant differences in the consumption of vitamins, maternal infections, and medication consumption (*p* < 0.05). The other factors evaluated (i.e., exposure to pollutants, diseases during pregnancy, maternal addictions) showed no significant differences. The specific environmental risk factors analyzed in mothers of individuals with CHD are represented in Supplementary Table S1.

### 3.2. DNA Methylation Levels of TBX20 Gene Promoter in the Study Groups

**Figure 1:**
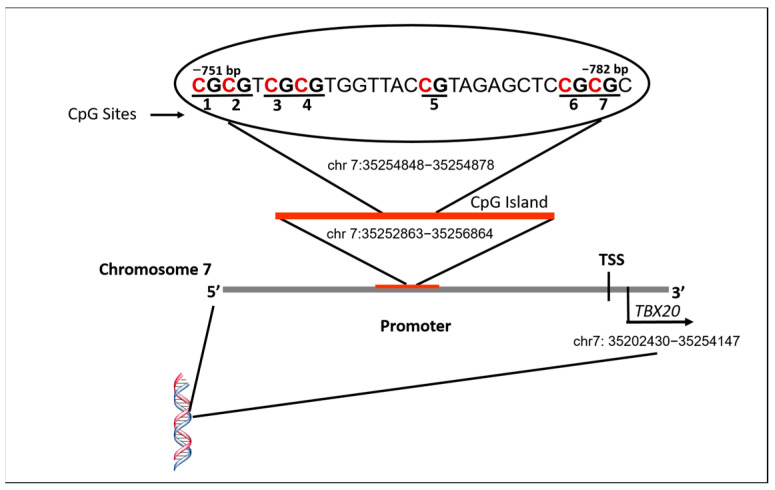
Illustration of the DNA sequence analyzed by pyrosequencing in *TBX20* gene promoter. CpG sites are shown with numbers 1–7. TSS, transcription start site.

**Figure 2:**
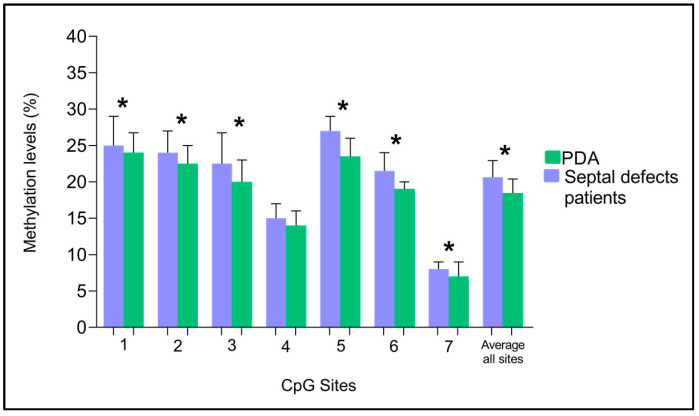
Analysis of the percentage of *TBX20* gene methylation in the study groups. The percentage of methylation is represented as medians and interquartile ranges. * *p* < 0.05, Mann-Whitney U test.

We analyzed seven CpG sites of the *TBX20* gene localized in the promoter region, which is described in Figure 1. In Figure 2, the DNA methylation levels of the *TBX20* gene promoter in the study groups are depicted. We found statistically significant differences between PDA and septal defect groups. The septal defects group showed higher methylation levels in comparison with the PDA group in all CpG sites, with the exception of CpG site 4. For the CpG site 1: 25% [IQR: 24–29%] vs. 24% [IQR: 21–26.75%], *p* = 0.002; CpG site 2: 24% [IQR: 22–27%] vs. 22.5% [IQR: 20.25–25%], *p* = 0.016; CpG site 3: 22.5% [IQR: 19.25–26.75%] vs. 20% [IQR: 18–23%], *p* = 0.001; CpG site 5: 27%[IQR: 23–29%] vs. 23.5% [IQR: 21–26%], *p* < 0.001; CpG site 6: 21.5% [IQR: 19–24%] vs. 19%[IQR: 17–20%], *p* < 0.001; CpG site 7: 8% [IQR: 7.25–9%] vs. 7%[IQR: 6–9%], *p* < 0.001; and the average of all CpG sites: 20.64% [IQR: 18.85–22.92%] vs. 18.46% [IQR: 16.85–20.39%], *p* < 0.001.

### 3.3. Association between DNA Methylation Levels of TBX20 Gene Promoter and the Risk of Septal Defects

The stratification of DNA methylation levels of the study groups into quartiles allowed us to identify a significant risk of septal defects development (OR = 4.73, 95% CI = 1.69–13.27, *p* = 0.003) in the highest quartiles of the average of all CpG sites. Afterward, the model was adjusted for maternal infections, and consumption of vitamins the risk association remained (OR = 4.59, 95% CI = 1.57–13.44, *p* = 0.005). The association data of methylation levels of each CpG site showed similarly a significantly increased risk of septal defects development in the highest quartile; CpG site 1: (OR = 5.26, 95% CI = 1.74–15.85, *p* = 0.003); CpG site 2: (OR = 2.94, 95% CI = 1.09–7.93, *p* = 0.033); CpG site 3: (OR = 5.41, 95% CI = 1.84–15.89, *p* = 0.002); CpG site 5: (OR = 4.04, 95% CI = 1.40–11.62, *p* = 0.010); CpG site 6: (OR = 9.86, 95% CI = 2.93–33.20, *p* < 0.001); and CpG site 7: (OR = 4.51, 95% CI = 1.22–16.63, *p* = 0.024); with the exception of CpG site 4: (OR = 2.03, 95% CI = 0.97–8.79, *p* = 0.055). Data are described in Table 2.

### 3.4. A Receiver Operator Characteristic (ROC) Curve Analysis of DNA Methylation Levels of TBX20 Gene Promoter

**Figure 3:**
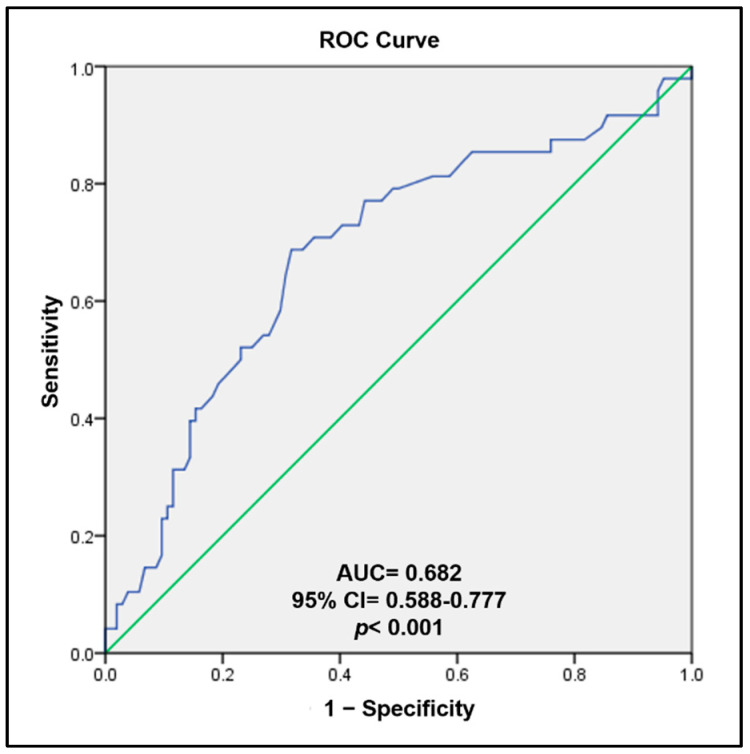
Receiver operator characteristic (ROC) curve as predictor value for the presence of septal defects by DNA methylation levels of *TBX20* gene promoter.

The ROC curve analysis showed an AUC of DNA methylation levels of *TBX20* gene promoter in patients with septal defects statistically significant (AUC = 0.682, 95% CI = 0.588–0.777, *p* < 0.001), Figure 3.

### 3.5. Association between Environmental Risk Factors and the Risk of Septal Defects Development

The association analysis of environmental risk factors during pregnancy in mothers of patients with septal defects, compared to the PDA group, showed that the consumption of vitamins was associated with a decreased risk (OR = 0.10; 95% CI = 0.01–0.98; *p* = 0.048) of the disease, while maternal infections were associated with an increased risk of septal defects (OR = 3.10; 95% CI = 1.26–7.60; *p* = 0.013). Medication consumption showed a trend toward an increased risk; however, this was not statistically significant. The other factors did not show any association (Table 3).

### 3.6. Analyze of Interaction of TBX20 Gene Methylation with Environmental Factors by Multifactor Dimensionality Reduction (MDR) Algorithm

Based on the quartiles of *TBX20* gene methylation levels, we further used MDR to analyze the possible interaction of the methylation with environmental factors. The analysis revealed two models suggesting interaction: *TBX20*-Methylation/Exposure to pollutants/Sex/Age, and *TBX20*-Methylation/Exposure to pollutants/Maternal infections/Sex/Age, with a Testing Accuracy of 0.6852 and 0.6442, respectively, and CVC of 10/10 in both models (*p* = 0.002 and 0.03, respectively) (Table 4). As the former model showed the highest testing accuracy was chosen as the best interaction model.

### 3.7. In Silico Analysis to Predict Transcription Factor Binding Sites in TBX20 Gene Promoter

The in silico analysis through bioinformatic tools showed binding sites for Hey-like basic Helix-Loop-Helix transcription factors (*bHLH*) and Specificity protein 1 (*Sp1*) transcription factors.

### 3.8. Identification of PPI Network in Cardiac Genes

**Figure 4:**
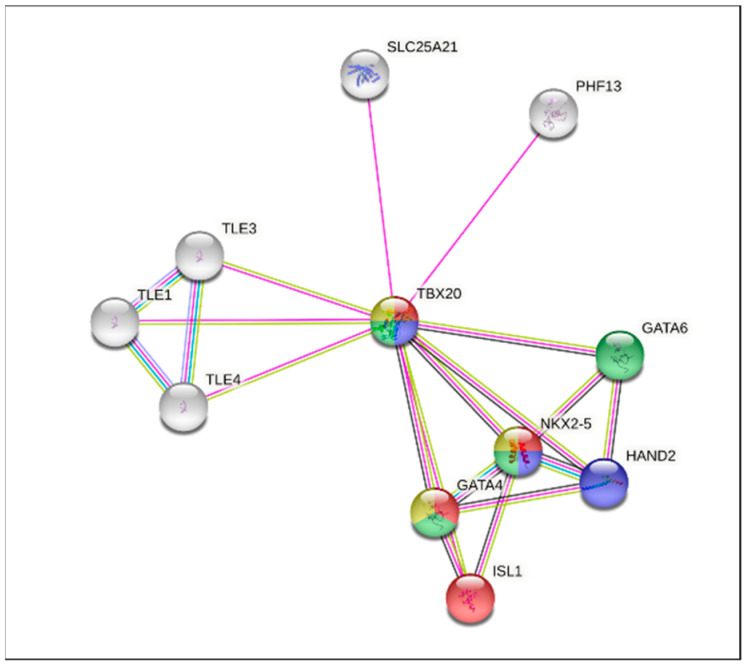
Protein-protein interaction (*PPI*) network of cardiac genes reported in CHD. The functional enrichments of this network showed the following results: (a) biological process: marked in blue, cardiac chamber formation (TBX20, NKX2.5, HAND2); marked in red, atrial septum formation (TBX20, GATA4, ISL1, NKX2.5); (b) disease gene associations: marked in green, atrial septal defect (TBX20, GATA4, GATA6, NKX2.5); marked in yellow, ventricular septal defect (TBX20, GATA4, NKX2.5).

The in silico analysis of this network reported the interaction between proteins SL25A21, PHF13, TLE3, TLE1, TLE4, TBX20, GATA6, GATA4, NKX2.5, HAND2, and ISL1. The functional enrichments of this network showed the following results: (a) biological process: the cardiac chamber formation is marked in blue, and the atrial septum formation is marked in red; (b) disease gene associations: the atrial septal defect is marked in green and the ventricular septal defect is marked in yellow (Figure 4).

### 3.9. Correlation Analysis between DNA Methylation Levels of TBX20 Gene Promoter and Age

**Figure 5:**
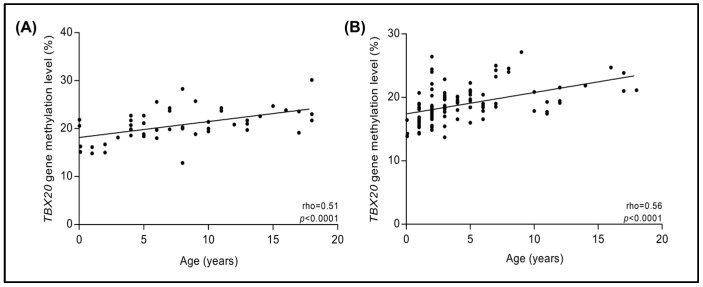
Correlation between DNA methylation levels of *TBX20* gene promoter and age. (**A**) Patients with septal defects; (**B**) PDA. *p* < 0.05, Spearman correlation.

A correlation analysis was conducted between DNA methylation levels of *TBX20* gene promoter and age by study groups, and the result showed a moderate positive correlation in the septal defect group as well as in the PDA group (rho = 0.51 and 0.56, respectively; *p* < 0.0001) (Figure 5). Likewise, we analyzed the correlations in the study groups by sex, observing a positive correlation in females (rho = 0.48, *p* = 0.013) but not in male patients with septal defects (rho = −0.05, *p* = 0.817). Similarly, a positive correlation was found in females and in males (rho = 0.53 and 0.60; respectively, *p* < 0.0001) of the PDA group. (Supplementary Figure S1).

### 3.10. Association between DNA Methylation Levels of TBX20 Gene Promoter and Sex

**Figure 6:**
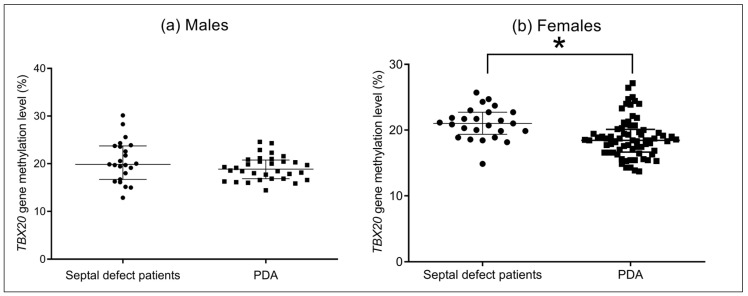
Association between DNA methylation levels of *TBX20* gene promoter by sex. (**a**) Males; (**b**) Females. * *p* < 0.001. Mann-Whitney U test.

A significant difference was found between the median value of average all sites of DNA methylation levels in female patients with septal defects and the female PDA group [21.0% (19.35–22.71%) vs. 18.42% (16.57–20.17%), *p* < 0.001]; nonetheless, the comparison between male patients with septal defects and the male PDA group did not show a significant difference [19.85% (16.71–23.71%) vs. 18.42% (16.57–20.17%), *p* = 0.236] (Figure 6). The CpG sites 1-7 also showed significant differences between female patients with septal defects and PDA females (*p* < 0.05); when males with septal defects were compared to PDA males, no significant difference was observed (*p* > 0.05). Data are described in Supplementary Table S2.

## 4. Discussion

During the cardiogenesis process, the participation of several transcription factors in heart formation is reported [31]. In the transcriptional regulation of cardiac genes, genetic and epigenetic mechanisms are implicated [32], such as mutations, microRNAs (miRNAs), and DNA methylation, all playing a critical role in cardiogenesis and are proposed as potential biomarkers for early identification of CHD [33].

In this study, we found evidence of an epigenetic mechanism, DNA methylation of *TBX20* gene promoter, influencing the development of septal defects. In addition, the analysis of environmental risk factors suggests a possible relation between the consumption of vitamins and maternal infections with septal defects. The analysis of interaction using MDR showed models suggesting an interaction between *TBX20* methylation, exposure to pollutants, maternal infections, sex, and age. 

On the other hand, the results of the bioinformatics analysis showed binding sites for *bHLH* and *Sp1* transcription factors, as well as a PPI network, where the TBX20 protein interacts with proteins encoded by cardiac genes, therefore, exhibiting an important role in the biological processes involved in the heart formation and gene-associated diseases such as ventricular and septal defects. 

*TBX20* is one of the primary genes involved in heart development; to date, however, its epigenetic mechanisms in CHD are not fully understood. During heart formation, the *TBX20* gene has a key role in the regulation and proliferation of cardiac cells, as well as in the formation of cardiac chambers [34,35]. This gene also exhibits activator and repressor transcriptional activities in transcription factors involved in heart development [12,13,14].

We observed high DNA methylation levels in patients with septal defects compared to the PDA group. These findings are in accordance with the study by González-Peña et al.; they evaluated the methylation status of the *TBX20* gene in patients with ventricular septal defects (VSD) and found higher methylation levels in the patient group than in the control group [18]. That study only included 16 children with VSD and 32 controls.

Our study, on the other hand, included a larger number of individuals in each group; additionally, we evaluated several environmental risk factors associated with CHD, while González-Peña et al. focused on the maternal dietary intake of folic acid. It is important to note that the experimental strategy used in both studies to determine the methylation levels were different. We used the pyrosequencing process, and González-Peña et al. used the Quantitative PCR (qPCR). Although both studies analyzed different regions of the *TBX20* gene promoter, we found a similar pattern of methylation with higher levels in the septal defects groups than in the comparison groups; therefore, the region near the transcription start site could represent an important regulator of transcriptional activity of *TBX20* gene [36]. To confirm these findings, more functional studies are required to understand better the epigenetic role of the *TBX20* gene in the development of CHD. 

Previous studies have evaluated the methylation status of the *TBX20* gene in other types of CHD, specifically in tetralogy of Fallot (TOF). TOF is caused by a single initial alteration, consisting of hypoplasia of the infundibular septum, characterized by interventricular communication, obstruction of the right ventricular outflow tract override of the ventricular septum by the aortic root, and right ventricular hypertrophy [37,38]. In the report, Gong et al. analyzed the methylation status of the *TBX20* gene in eight patients with TOF and five controls and reported higher methylation levels in controls [19]. Yang et al. investigated the association between the methylation levels of the *TBX20* gene in 23 patients with TOF and 5 controls, finding lower methylation levels in TOF [39]. Additionally, Sheng et al. evaluated the methylation status in 31 patients with TOF and 13 controls; similarly, they found higher methylation levels in the control group [40]. In brief, these authors found lower methylation levels in patients with TOF, while we reported the opposite data. A possible explanation of these results involves the study population and the type of CHD that might affect the methylation status.

The ROC curve analysis showed a discriminatory capacity to predict the presence of septal defects by analyzing the methylation levels of the *TBX20* gene promoter. These results suggest that high DNA methylation levels of the *TBX20* gene could be a good strategy for assessing the risk of septal defects.

In addition, we identified differences in methylation levels that might be associated with septal defect development. The methylation status in the promoter region could lead to transcriptional repression and disrupt the gene expression and function of the *TBX20* gene [41,42]. Exposure to different environmental risk factors during gestation could affect organogenesis and lead to structural abnormalities [43]; regarding CHDs, several studies have reported environmental risk factors, including gestational diabetes, obesity, medication consumption, infections, and maternal addiction [24].

Our analysis of environmental risk factors suggests that the consumption of vitamins, such as folic acid and ferrous fumarate, could be protective factors against septal defects. This agrees with the literature: in a meta-analysis, Feng et al. evaluated epidemiological observational studies of folic acid consumed by pregnant women before becoming pregnant and the risk of CHDs in their offspring, finding robust evidence that maternal folic acid (FA) intake reduces the risk of CHDs [44]. Similarly, Mao et al. investigated the association between maternal FA supplementation and the risk of CHDs in a birth cohort study. Based on their results, they suggested that FA intake before pregnancy was associated with a reduced risk of CHDs [45].

Regarding maternal intake of FA, González-Peña et al. also evaluated mothers of patients with VSD and a control group. They found a relationship between maternal intake FA and the methylation status of the *TBX20* gene associated with VSD, reporting that a higher methylation status of the *TBX20* gene was associated with FA maternal intake [18]. On the other hand, some studies have reported the association of maternal infections with CHDs. García-Flores et al. observed that maternal infections increased the risk of CHDs [46]. Similarly, Dolk et al. also reported that maternal infections (vaginal infections) were increased in mothers of children with CHDs based on a case-control study [47]. Nevertheless, to date, there is no evidence of the mechanism by which maternal infections regulate or participate in CHDs. More studies are needed to confirm these findings. It would be interesting to evaluate these environmental factors in a prospective cohort during pregnancy and after birth to consider bias/confounding factors.

The association of environmental factors with CHD agrees with previous reports; however, nowadays, the molecular mechanisms of regulation of these environmental factors in the etiology of CHD are not clear. In our study, the MDR analysis identified a significant interaction between *TBX20* methylation, exposure to pollutants, age, and sex, which could be evidence of a necessary interplay of environmental factors with epigenetic mechanisms, such as DNA methylation, in the development of CHD [48,49,50,51,52].

Additionally, the MDR analysis showed an interaction between *TBX20* methylation with age. This result agrees with the finding of a correlation between methylation levels of *TBX20* and age since we observed a moderate increase in methylation levels according to age in the septal defect group as well as in the PDA group. Previous studies have described this phenomenon, indicating that the CpG sites distributed along the whole human genome could present dynamic changes according to age [53,54,55]. Nevertheless, further research is necessary to clarify the relationship and interaction between age, environmental factors, and methylation. 

The bioinformatics analysis to identify binding sites in the analyzed sequence showed binding sites for Hey-like basic Helix-Loop-Helix (*bHLH*) transcription factor and Specificity protein 1 (*Sp1*). *bHLH* transcription factors are transcriptional repressors, while Hey proteins participate in cardiomyocyte differentiation [56] and regulate a critical process for valve and septum formation [57]. It has been shown that mice deficient in the Hey1/L gene develop CHD leading to heart failure soon after birth [58]. Sakata et al. reported that Hey2 plays a role in the regulation of ventricular septation in mammalian heart development, and it is important for normal myocardial contractility [59]. Our findings suggest that this transcription factor could participate in the regulation of the *TBX20* gene; however, this hypothesis should be validated in functional studies with another regulatory mechanism to confirm these findings. 

*Sp1* is a transcription factor member of the Sp/Kruppel-like factor (*KLF*) family, having a fundamental role in different biological processes, with a relevant function in embryonic development, cell cycle regulation, and cell differentiation; *Sp1* has been implicated in several diseases [60,61]. *Sp1* presents binding sites for GC- or GT-boxes. It has the capacity to promote and activate transcription in the promoter region of eukaryotes [62,63] and to interact with other transcription factors, corepressors, and coactivators to modulate gene expression [49].

This factor has been reported previously in CHDs. García-Flores et al. observed binding sites for *Sp1* in *TBX5* gene promoter analyzed by pyrosequencing in patients with septal defects [46]. Gong J et al. also evaluated a region of the *TBX20* gene in patients with Tetralogy of Fallot and found a hypomethylation in the *TBX20* gene promoter region in the patient group. They reported that high expression of the *TBX20* gene could be caused by *Sp1* binding at the region analyzed due to the decreased methylation at the *Sp1* transcription factor binding sites [19]. Li et al. investigated the expression profile in *Nkx2.5* knock-out embryonic mice and constructed a transcriptional regulatory network to identify differentially expressed genes (DEGs) and reported a down-regulation of *Sp1* among other transcription factors; they concluded that DEGs could provide new knowledge to understand the mechanisms implicated in the development of CHDs [64]. These results indicate the relevance of *Sp1* having a possible key role in regulating the transcription of genes that participate in the pathogenesis of CHDs. 

Thus, the use of bioinformatics tools is an excellent strategy for performing analysis by computer simulations and providing experimental data to explain the mechanisms involved in different diseases. In this study, the protein-protein interaction network for the TBX20 protein identified, in the functional enrichments analysis, its interaction with several transcription factors (*Nkx2.5*, *GATA4*, *GATA6*, *HAND2,* and *ILS1*) that are associated with heart formation and CHDs [65,66,67,68]. The literature confirms the different interactions found in our study, where different transcription factors participate in several transcriptional pathways involved in the cardiogenesis process [9,31,66].

Regarding the difference by sex in the methylation levels that we found, the literature indicates that neuro-hormonal, genetic, and morphological factors could have an effect on the incidence of women with CHD, inducing a possible alteration in the methylation status [69,70]. Sex differences in CHD should be evaluated in additional studies to confirm our findings.

Our study has several strengths: (a) we assessed the effect of being exposed to different environmental risk factors during pregnancy in mothers of individuals with CHDs on the methylation levels of *TBX20*, (b) we evaluated the DNA methylation levels by a quantitative experimental strategy: pyrosequencing, (c) we used bioinformatic tools to perform in silico analyses, where the computational modeling included the prediction of bindings sites for transcription factors and the development of protein-protein interaction network for the TBX20 protein.

Our study also has some limitations that should be mentioned: (a) we analyzed a region belonging to one CpG Island, additional CpG Islands should be evaluated in order to identify the methylation status of the promoter region, (b) we did not perform a functional study to validate the effect of the methylation status on the regulation of transcriptional activity of *TBX20* gene, (c) we could not perform a genetic investigation such as array-CGH or NGS panels to discard the presence of mutations in genes associated with CHD; nevertheless, this assessment was conducted by clinical and phenotypic examination performed by two experienced medical geneticists who discarded any syndromic association, d) we have a limited number of participants. Thus, additional studies in other populations with different genetic backgrounds and with larger samples of CHD are required to confirm our findings.

## 5. Conclusions

Our findings suggest that high DNA methylation levels of the *TBX20* gene promoter are associated with congenital septal defects. Moreover, we identified some environmental factors that can be used as protective and risk markers for developing congenital heart defects. These findings contribute to new knowledge in the epigenetic field involved in the pathogenesis of congenital septal defects. Further studies should be performed to confirm our findings.

## Figures and Tables

**Table 1:** General characteristics of the study population.

	PDA (*n* = 104)	Septal Defects Patients (*n* = 48)	* p
**Sex (%)**			0.041
Men	32 (30.8)	23 (47.9)	
Women	72 (69.2)	25 (52.1)	
**Age (years)**	3 (2–6)	7 (4–11.7)	0.0001

* *p* < 0.05, Mann-Whitney U or Chi-squared test.

**Table 2:** Association between DNA methylation levels of *TBX20* gene promoter and the risk of septal defects.

		Crude Model		Model 1	
Quartile	**Methylation (%)**	**OR (95% CI)**	* p	**OR (95% CI)**	* *p*
	**Average of All Sites**				
1	≤17.42	1		1	
2	17.43–19.14	0.80 (0.24–2.66)	0.721	0.681 (0.19–2.32)	0.540
3	19.15–21.14	2.60 (0.90–7.51)	0.076	3.24 (1.06–9.88)	0.039
4	≥21.15	4.73 (1.69–13.27)	0.003	4.59 (1.57–13.44)	0.005
	**CpG Site 1**				
1	≤22	1		1	
2	22.1–24	1.58 (0.51–4.91)	0.427	1.74 (0.54–5.58)	0.352
3	24.1–27	3.57 (1.27–10.02)	0.015	4.16 (1.43–12.10)	0.009
4	≥27.1	4.60 (1.62–13.03)	0.004	5.26 (1.74–15.85)	0.003
	**CpG Site 2**				
1	≤21	1		1	
2	21.1–23	1.35 (0.47–3.86)	0.569	1.17 (0.38–3.54)	0.776
3	23.1–25	2.37 (0.92–6.09)	0.073	2.88 (1.08–7.73)	0.035
4	≥25.1	2.94 (1.13–7.60)	0.026	2.94 (1.09–7.93)	0.033
	**CpG Site 3**				
1	≤18	1		1	
2	18.1–20	1.21 (0.39–3.76)	0.740	1.14 (0.35–3.77)	0.827
3	20.1–24	2.50 (0.96–7.02)	0.060	3.14 (1.12–8.78)	0.029
4	≥24.1	5.06 (1.82–14.03)	0.002	5.41 (1.84–15.89)	0.002
	**CpG Site 4**				
1	≤13	1		1	
2	13.1–15	1.55 (0.62–3.84)	0.339	1.55 (0.61–3.93)	0.353
3	15.1–17	1.59 (0.60–4.18)	0.344	1.85 (0.67–5.08)	0.232
4	≥17.1	3.33 (1.16–9.58)	0.025	2.03 (0.97–8.79)	0.055
	**CpG Site 5**				
1	≤22	1		1	
2	22.1–24	0.87 (0.27–2.80)	0.815	0.88 (0.26–2.98)	0.881
3	24.1–28	3.00 (1.22–7.37)	0.017	3.25 (1.29–8.22)	0.012
4	≥28.1	4.30 (1.58–11.47)	0.004	4.04 (1.40–11.62)	0.010
	**CpG Site 6**				
1	≤17	1		1	
2	17.1–19	1.46 (0.45–4.68)	0.523	1.52 (0.46–4.97)	0.483
3	19.1–22	3.32 (1.15–9.54)	0.026	3.18 (1.07–9.46)	0.037
4	≥22.1	9.63 (2.99–30.96)	0.0001	9.86 (2.93–33.20)	0.0001
	**CpG Site 7**				
1	≤6.75	1	1		
2	6.76–8	2.97 (1.00–8.77)	0.048	2.96 (0.98–8.94)	0.054
3	8.1–9	5.54 (1.67–18.41)	0.005	6.66 (1.89–23.37)	0.003
4	≥9.1	5.33 (1.51–18.84)	0.009	4.51 (1.22–16.63)	0.024

OR:odds ratio, 95% CI: confidence intervals. Model 1: adjusted by maternal infections and consumption of vitamins. * *p* < 0.05, Logistic regression.

**Table 3:** Association of environmental risk factors with the study population.

Environmental Risk Factor	OR (95% CI)	* p
Exposure to pollutants	1.09 (0.54–2.20)	0.806
Consumption of vitamins	0.10 (0.01–0.98)	0.048
Diseases during pregnancy	1.23 (0.58–2.60)	0.582
Maternal infections	3.10 (1.26–7.60)	0.013
Maternal addictions	0.81 (0.34–1.92)	0.637
Medication consumption	1.81 (0.91–3.62)	0.090

OR, odds ratio, CI, confidence interval. * *p* < 0.05, Univariate logistic regression.

**Table 4:** Interaction of *TBX20* gene methylation with environmental factors in CHD.

Model	Training Accuracy	Testing Accuracy	CVC	* p
*TBX20*-Methylation, Sex, Age	0.8896	0.5801	7/10	0.31
*TBX20*-Methylation, Exposure to pollutants, Sex, Age	0.9435	0.6852	10/10	0.002
*TBX20*-Methylation, Exposure to pollutants, Maternal infections, Sex, Age	0.9701	0.6442	10/10	0.03
*TBX20*-Methylation, Exposure to pollutants, Consumption of vitamins, Maternal infections, Sex, Age	0.9797	0.6346	8/10	0.04

* p < 0.05, Chi-squared test.

## Data Availability

Data supporting results are available from the corresponding authors upon reasonable request.

## Supplementary Materials

The following supporting information can be downloaded at: https://www.mdpi.com/article/10.3390/cells12040586/s1, Table S1: Environmental risk factors during pregnancy in mothers of individuals with CHD; Table S2: Analysis of the percentage of methylation of *TBX20* gene in the study groups by sex; Figure S1: Correlation of DNA methylation levels of *TBX20* gene promoter and age by sex. (A) Men, septal defects patients; (B) Women, Septal defects patients; (C) Men, PDA; (D) Women, PDA. Spearman correlation.
