# Supplementary material for: Epigenetic Evaluation of the TBX20 Gene and Environmental Risk Factors in Mexican Paediatric Patients with Congenital Septal Defects
Source: Cells. 2023 Feb 11;12(4):586. doi: 10.3390/cells12040586 (PMC9953838; doi:10.3390/cells12040586)
Supplement: Supplementary file 1 [file cells-12-00586-s001.zip › cells-2091588-supplementary.pdf]

Supplementary Table S1. Environmental risk factors during pregnancy in mothers of individuals with CHD.

Environmental factors	PDA (*n*=104)	Septal defects Patients (*n*=48)	*p**
**Exposure to pollutants (%)**			0.824
Heavy metals	6 (5.8)	5 (10.4)	
Pesticides	15 (14.4)	4 (8.3)	
Solvents	6 (5.8)	7 (14.6)	
Herbicides	2 (4.2)	1 (2.9)	
Varnishes	6 (5.8)	0	
Unknown	0	0	
No exposure	65 (62.5)	29 (60.4)	
**Consumption of Vitamins (%)**			0.006
Folic Acid/Ferrous Fumarate	103 (99)	44 (91.7)	
Unknown	0	4 (8.3)	
No exposure	1 (1)	0	
**Diseases during pregnancy (%)**			0.246
Preeclampsia	7 (6.7)	6 (12.5)	
Hypertension	8 (7.7)	3 (6.3)	
Gestational diabetes	2 (1.9)	3 (6.3)	
Diabetes mellitus type 1	2 (1.9)	1 (2.1)	
Diabetes mellitus type 2	2 (1.9)	2 (4.2)	
Epilepsy	1 (1)	0	
Rheumatoid arthritis	3 (2.9)	0	
Preeclampsia/DM T2	1 (1)	0	
H/DM T1	1 (1)	0	
DM T2/RA	1 (1)	0	
Unknown	2 (1.9)	0	
No exposure	74 (71.2)	33 (68.8)	
**Maternal infections (%)**			0.016
Urinary tract infection	45 (43.3)	19 (39.6)	
Vaginal candidiasis	16 (15.4)	14 (29.2)	
UTI/VC	8 (16.7)	7 (6.7)	
Unknown	0	0	
No exposure	36 (34.6)	7 (14.6)	
**Maternal addiction (%)**			0.485
Alcoholism	8 (7.7)	2 (4.2)	
Smoking	11 (10.6)	7 (14.6)	
Alcoholism /Smoking	4 (3.8)	0	
Unknown	0	0	

No exposure	81 (77.9)	39 (81.3)	
**Medication consumption (%)**			0.028
Nitrofurantoin	7 (6.7)	7 (14.6)	
Nitrofurantoin/Insulin	0	1 (2.1)	
Nitrofurantoin/Ampicillin	0	2 (4.2)	
Nitrofurantoin/ Nystatin	1 (1.0)	0	
Ampicillin	8 (7.7)	3 (6.3)	
NSAIDs	3 (2.9)	20	
NSAIDs/Nitrofurantoin	5 (4.8)	2 (4.2)	
Alfametildopa	7 (6.7)	3 (6.3)	
Alfametildopa/Ampicilin	5 (4.8)	2 (4.7)	
Alfametildopa/Hydralazine	2 (1.9)	0	
Alfametildopa/Nitrofurantoin	0	2 (4.2)	
Alfametildopa/Dexamethasone	0	2 (4.2)	
Nystatin	8 (7.7)	3 (6.3)	
Unknown	0	0	
No exposure	63 (60.3)	22 (45.8)	

Data are expressed as percentages. *Chi-squared test. DM T2, Diabetes mellitus type 2; H, Hypertension; DM T1, Diabetes mellitus type 1; NSAIDs, Nonsteroidal anti-inflammatory drugs; VC, Vaginal candidiasis; UTI, Urinary Tract Infection; RA, Rheumatoid arthritis.

Supplementary Table S2. Analysis of the percentage of methylation levels of the *TBX20* gene in the study groups by sex.

**CpG site**	**Females PDA (*n*= 72)**	**Female septal defects patients (*n*= 25)**	***p****	**Males PDA (*n*= 32)**	**Male patients with septal defects (*n*= 23)**	***p****
1	24 (22-26.25)	25 (24-28.5)	0.016	24 (21-27)	25 (23-33)	0.062
2	23 (20.75-25)	25 (22.5-27)	0.025	22 (20.25-24.75)	23 (21-27)	0.214
3	20 (18-23)	23 (21-26.5)	0.001	20.5 (18-24)	21 (18-28)	0.292
4	14 (13-16)	16 (15-17.5)	0.012	15 (13-16.75)	15 (12-17)	0.945
5	23 (20.75-25.25)	28 (24.5-28.5)	0.001	24 (22-26.75)	26 (22-29)	0.472
6	18 (16-20)	22 (20-24)	0.001	19 (17.25-21)	21 (17-25)	0.113
7	7 (6-9)	9 (8-9)	0.001	7 (7-8.75)	8 (7-9)	0.095
Average all sites	18.42 (16.57-20.17)	21 (19.35-22.71)	0.001	18.42 (16.57-20.17)	21 (19.35-22.71)	0.236

The percentage of methylation is represented as medians and interquartile ranges. * Mann–Whitney U test.

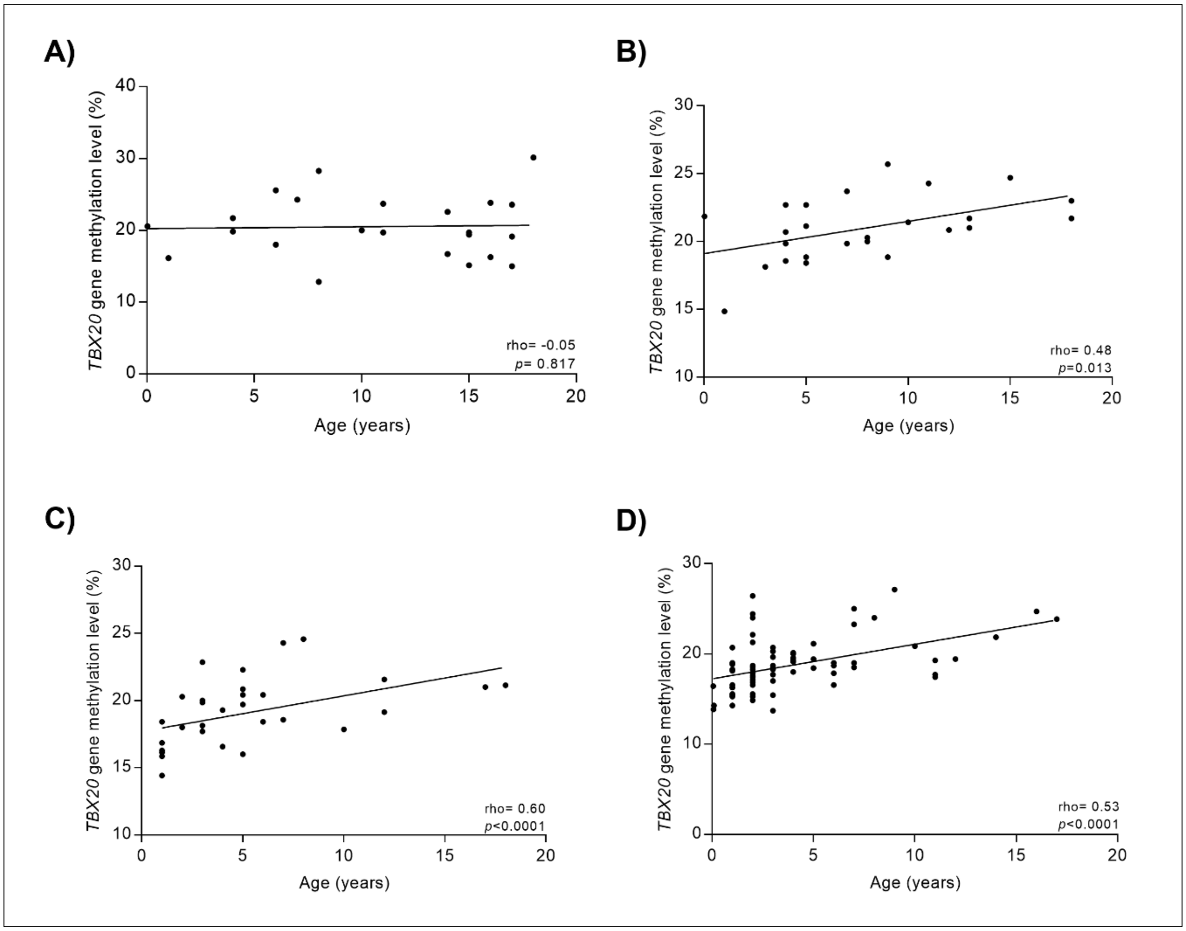

Supplementary Figure S1. Correlation of DNA methylation levels of the *TBX20* gene promoter and age by sex. A) Men, Septal defects patients; B) Woman, Septal defects patients; C) Men, PDA; D) Women, PDA. Spearman correlation.
